# Biotechnological Resources to Increase Disease-Resistance by Improving Plant Immunity: A Sustainable Approach to Save Cereal Crop Production

**DOI:** 10.3390/plants10061146

**Published:** 2021-06-04

**Authors:** Valentina Bigini, Francesco Camerlengo, Ermelinda Botticella, Francesco Sestili, Daniel V. Savatin

**Affiliations:** 1 Department of Agriculture and Forest Sciences, University of Tuscia, 01100 Viterbo, Italy; valentina.bigini@unitus.it (V.B.); f.camerlengo@unitus.it (F.C.); 2 Institute of Sciences of Food Production (ISPA), National Research Council (CNR), 73100 Lecce, Italy; ermelinda.botticella@cnr.it

**Keywords:** crop disease resistance, plant-microbe interaction, molecular mechanisms in plant immunity, sustainable agriculture

## Abstract

Plant diseases are globally causing substantial losses in staple crop production, undermining the urgent goal of a 60% increase needed to meet the food demand, a task made more challenging by the climate changes. Main consequences concern the reduction of food amount and quality. Crop diseases also compromise food safety due to the presence of pesticides and/or toxins. Nowadays, biotechnology represents our best resource both for protecting crop yield and for a science-based increased sustainability in agriculture. Over the last decades, agricultural biotechnologies have made important progress based on the diffusion of new, fast and efficient technologies, offering a broad spectrum of options for understanding plant molecular mechanisms and breeding. This knowledge is accelerating the identification of key resistance traits to be rapidly and efficiently transferred and applied in crop breeding programs. This review gathers examples of how disease resistance may be implemented in cereals by exploiting a combination of basic research derived knowledge with fast and precise genetic engineering techniques. Priming and/or boosting the immune system in crops represent a sustainable, rapid and effective way to save part of the global harvest currently lost to diseases and to prevent food contamination.

## 1. Introduction

With the new millennium, humankind is facing issues for too long postponed. Among the greatest challenges is to meet the food demand for a rapid increase in global population, estimated to exceed 9 billion by 2050 [1]. Additionally, climate changes negatively impact crop production as well as water and land availability for agriculture [2]. Cereals are cultivated for their edible caryopses in greater quantities worldwide and provide more food energy to humans than any other crop; wheat, maize and rice are the most important crops worldwide. 

Food availability and security challenge may be overcome by boosting crop yield, particularly that of cereals, and/or by reducing crop yield losses (20–40%) to pests and diseases, therefore diminishing further consequences for livelihoods, public health and the environment [3]. Usage of chemical pesticides is the most widely used method to eliminate or minimize the severity of diseases affecting crops. However, different studies are highlighting several negative side-effects of the long-term use of pesticides [4], such as toxicity in humans, effects on non-target organisms—pollinators and soil microbiota—with consequent damage to ecosystems, and pollution of water and soil systems [5]. Moreover, effectiveness of long-term use of pesticides is impeded by different levels of resistance developed by phytopathogens [6]. Crop rotation, aiming to prevent the pathogen accumulation by alternating an incompatible host, together with the introduction of plant disease resistance genes (*R* genes) through specific breeding programs, represents alternative methods to combat yield losses to pests. Notably, crop rotation is not always an economically viable strategy [7], whereas classical breeding programs are not applicable in some crops for which no resistant cultivars are available. In addition, pathogens can quickly overcome plant host resistance mechanisms, particularly when resistance is encoded by a single gene [8]. For example, rice cultivars that are resistant to *Magnaporthe oryzae* typically become ineffective every 2–3 years [9]. 

Due to the existing combination of these problems, food availability and safety continue to be an area of concern, with climate changes putting an ever-growing pressure on agriculture to search for further alternatives. Thus, sustainable yield increase, diminishing usage of chemicals and toxic compounds, enhancing crop resilience to biotic and abiotic stress and improving nutritional and healthiness values represent the main, concomitant, targets to be pursued in agriculture in the shortest period of time. In this scenario, it would be very difficult, if not impossible, to succeed with conventional breeding, and the role of plant sciences and biotechnology becomes crucial for the future of humankind. Therefore, to find harmless control strategies for crop disease management, we need to exploit the plant innate immunity that, if timely activated, can efficiently contrast and restrict plant infection by microorganisms. In fact, although in nature plants face many types of biotic stresses caused by various organisms including fungi, viruses, bacteria, nematodes and insects, they generally resist most pathogens, and plant infection is usually the exception, not the rule [10]. As sessile organisms, plants continuously monitor their living environments and modify, accordingly, their growth, development, and defense in order to better adapt and optimize reproductivity. Plants possess an innate ability to sense and recognize potential invading microorganisms and to mount successful defenses [10]. Only pathogens with an evolved ability to evade recognition or suppress host defense mechanisms, or both, are successful. These biotic stress agents cause different kinds of diseases, infections, and damage to cultivated plants and significantly impact crop productivity [11]. Particular attention is paid to fungal diseases, one of the most destructive groups of cereal crop pathogens and one which is favored by climate changes. They not only cause a reduction in both grain quantity and quality but can also be dangerous for human health due to the production of high concentrations of mycotoxins. Moreover, rice blast and wheat Fusarium Head Blight (FHB) or Take-all diseases can in some cases eliminate an entire cereal crop [12,13].

In this manuscript, we provide several examples of how existing biotechnological techniques can provide insights into gene function by adding, suppressing, or enhancing gene activities. Identification of key regulators involved in plant resistance/adaptation mechanisms, combined with available fast and precise biotechnological techniques, offers the potential to rapidly act on (a)biotic stress-derived yield losses, supporting crops to finally reach their full productivity in different and changing environments.

## 2. Plant Biotechnology: From Random to Directed, Precise and Safe Mutagenesis

**Figure 1:**
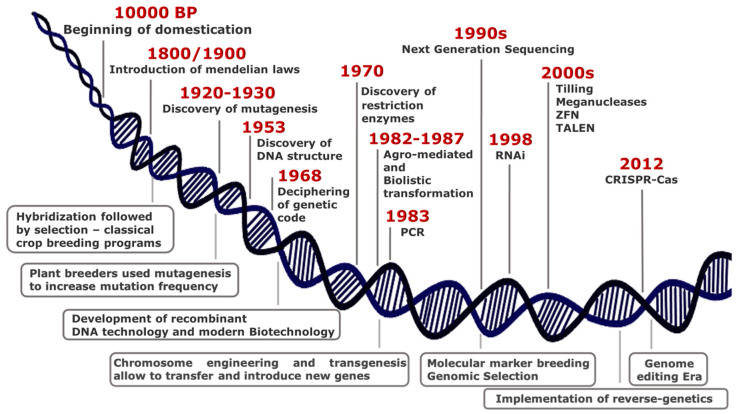
Agricultural biotechnology timeline. A timeline showing how biotechnology in agriculture has evolved, changing the ability to develop new crops.

Over thousands of years since 10,000 BP, humans have domesticated plants in an unconscious manner, selecting phenotypes with traits essential either for wide adaptation to different environments or improved agronomic performance. The phenotypic changes associated with adaptation under domestication pressure are referred to as “domestication syndrome” [14]. At the turn of 19th century, the introduction of Mendelian laws led to a scientific approach in crop breeding, thus representing the first revolution in the field of plant science (Figure 1).

Increased yield and abiotic and biotic resistance followed by enhanced performance in agronomical practices characterized early plant breeding programs by promoting the development of monotypic crop fields, with consequent loss of genetic variability.

The practice of hybridization followed by selection as a crop improvement strategy was initiated in the latter part of the 19th century by Vilmorin in France and by Wilhelm Rimpau in Germany in 1875 [15]. Different strategies of crossing permitted the increase of genetic variability useful to introduce desired traits in cultivars, leading to the most important modern crops [16].

One of the most important achievement that led to the green revolution was the harnessing of dwarf and semi-dwarf genes found in spontaneous or induced mutant wheats between 1950 and the late 1960s and introduced into modern cultivars by crosses [17]. 

Although the most common way of generating genetic variability is to mate (cross) two or more parents that have contrasting genotypes, the selection of best resulting phenotypes fostered the development of monotypic crop fields, with consequent loss of biodiversity.

Genetic variability is the basis to discover new beneficial traits and results from mutations that have occurred in genomes, either naturally or induced. Spontaneous mutations able to produce effects on phenotypes occur at low frequency in nature and the discovery of mutagenesis between 1920–1930 [18] allowed plant breeders to boost random mutation frequency by using chemical or physical mutation agents. Irradiation can cause deletions, inversion, and translocation besides point mutations, whereas the use of chemical agents strictly produces point mutations, especially transitions [19,20]. 

Plant breeders have used mutagenesis intensively since 1950, and to date, the FAO/IAEA Mutant Varieties Database includes more than 3300 varieties that have been released worldwide for commercial use, including more than 1500 cereal varieties.

The discovery of the DNA structure by Watson and Crick in 1953, the deciphering of genetic code in 1968, the finding of restriction enzymes in 1970 by Nathan, and the development of recombinant DNA technology in 1973 by Cohen and Boyer paved the way for the rise of modern plant biotechnology and molecular breeding (Figure 1) [21].

Some important achievements in plant sciences characterized the second half of the last century: the development of tissue culture and regeneration techniques allowing the use of embryo rescue and doubled haploid, and the genetic engineering technology including chromosome engineering and transgenesis for gene transfer between species distantly related. A further milestone in plant biotechnology was the demonstration that *Agrobacterium tumefaciens* Ti plasmid can be used to integrate foreign DNA into the plant genome [22,23,24]. Soon after, a direct gene transfer method, known as particle bombardment or biolistic, was established for recalcitrant monocots species, especially cereals [25,26]. Genetic manipulation quickly proved to have a great potential in functional genomics contributing to unravel essential in plant physiology mechanisms. In few years, transgenesis was widely adopted in plant breeding programs since it renders possible introgression of genes or any DNA sequence from other species and enables targeted editing of plant genome to increase genetic variability.

During 1990s, several genetic modified (GM) crops were developed and released on the market, with regulatory approvals of 44 countries reaching more than 400 GM events involving 32 different crops, among which were maize, rice and wheat. 

Among the most important traits introduced in GM crops are: (i) herbicide tolerance—introduced for the first time in soybean by Monsanto—that gave rise to Roundup Ready crops; (ii) biotic and abiotic stress resistance with the introduction of a gene encoding the crystal Bt toxin, providing protection against pests, firstly in soybean and thereafter in other crops like maize; (iii) improved yield and growth; (iv) product quality (the first GM crop released on the market was the Flavr Savr tomato that slow down the fruit softening) [27]; (v) biofortification (the most prominent example is the Golden Rice, in which an entire biosynthetic pathway has been introduced into rice to produce beta-carotene in the endosperm) [28]; (vi) pharmaceuticals, *in planta* production of molecules and development of edible vaccines; (vii) phytoremediation [29]. 

The last 30 years have been full of new discoveries in the area of plant biotechnology, which are more and more used by plant scientists and breeders (Figure 1). 

Starting from the 1990s, molecular markers assist in plant selection, since many genetic markers associated with loci controlling traits of agronomic interest provided the opportunity to accelerate gain from selection. The development of next-generation sequencing technologies opened the era of Genomic Selection (GS), which allowed simultaneous selection for numerous markers, estimating the effects of all together loci at phenotypic level that would otherwise have no significant effect if individually taken. GS combined with high-throughput phenotyping became a powerful tool for the selection of the best phenotype within a plant population and to discover genes associated with quantitative traits. Several resistance genes have been isolated in potato [30], wheat [31], rice [32,33], and barley [34].

In the 2000s, a new approach, the Targeting Induced Local Lesions in Genomes (TILLING) approach, was introduced to identify mutant genotypes harboring mutations in genes of interest [35]. This technique allowed researchers to obtain commercial non-transgenic, powdery-mildew-resistant bread wheat varieties [36].

In the last decades, new breeding techniques (NBTs) are rapidly emerging from advances in genomic research and for application in crop traits improvement. They enable precise, targeted, and reliable changes in the genome and do not create multiple, unknown, unintended mutations, unlike chemical or radiation-induced mutagenesis.

Genome-editing methods produce defined mutants, thus becoming a potent tool in functional genomics and crop breeding. Zinc Finger Nucleases (ZFN) and Transcription Activator-Like Effector Nucleases (TALENs) were the dominant genome editing tools until the rise of Clustered Regularly Interspaced Short Palindromic Repeats (CRISPR) and Crispr associated protein (Cas). CRISPR-Cas is an antiviral system developed by bacteria: segments of DNA containing short, repetitive base sequences (crispr RNA, crRNA) conserve the memory of intruding nucleic acids. The system is composed of genes encoding Cas nucleases, such as Cas9, and unique spacers (sequences complementary to a target genomic sequence) located in a genomic locus forming the CRISPR array along with crRNAs and trans-activating crRNA (tracrRNA), which are partially complementary to crRNAs. The RNase III processes the transcribed mRNA, releasing crRNA/tracrRNA complexes that activate and guide Cas proteins to target specific genomic loci introducing double strand breaks [37]. For the first time ever, researchers and breeders can select and target any location in the genome by the use of a short synthetic guide RNA (sgRNA) along with an endonuclease enzyme (Cas9) [38]. Due to high editing efficiency, multiplex editing capability and ease of usage, CRISPR technologies were quickly adopted for various genome-targeting purposes. For several genome-editing techniques, the resultant plants are free from foreign genes and would be indistinguishable both from plants generated by conventional breeding techniques and from naturally mutated plants. Thus, it is difficult for the plant scientific community, especially in Europe, to understand and accept the reasons why the European Court of Justice has recently restricted (almost forbidden) the infield growth of plants obtained by precision breeding techniques like CRISPR.

To date, major and minor crops, dicots and monocots, have been edited to improve traits of agronomical interest and with an increasing attention to nutritional and healthy values of derived foods [39]. Yield remains the major concern in crop breeding; the *Gn1a*, *DEP1* and *GS3* genes were edited in rice to enhance grain number and grain size [40]; knock-out mutations in wheat *Grain Weight 2* (*GW2*) gene increases grain weight and yield [41,42]. Improvement of resistance to biotic and abiotic stresses has also been achieved through genome editing technologies. The simultaneous modification of the three homoeologs of *EDR1* in wheat results in plants resistant to powdery mildew [43]; rice lines with broad-spectrum resistance to Xanthomonas have been produced by editing the promoter regions of *SWEET11*, *SWEET13,* and *SWEET14* genes [44].

Food nutritional quality and safety are essential prerogatives to feed burgeoning world population and to limit malnourishment. Waltz (2016) [45] knocked out gene encoding for polyphenol oxidase (PPO), producing a non-browning mushroom; Sun et al. [46] produced high-amylose rice through targeted mutations in the *SBEIIb* gene; recently, DuPont Pioneer announced intentions to commercialize waxy maize obtained by knock-out of *Wx1* gene [47]; the production of low immunogenic foods has been achieved by editing gliadin genes involved in celiac disease [48] and by editing α-amylase/trypsin inhibitors in wheat [49].

Genome editing techniques have also been used to accelerate the domestication of crops [50] or to create herbicide-resistant crops [51]. CRISPR-Cas technologies are constantly developing to overcome some limitations such as off-target effects, restrictive protospacer adjacent motif (PAM) sequences, and the low efficiency of homologous recombination. The discovery of new Cas9 orthologs (Cpf1, Cas13) and the introduction of prime editing by fusing Cas9 to reverse transcriptase [52] enable to extend genome editing applications. CRISPR editors represent a new genome editing approach for producing precise point mutations; nickase Cas9 (nCas9) fused to an enzyme (cytidine deaminase or adenosine deaminase) with base conversion activity, can convert one nucleotide into another [53,54]. Gene regulation can be achieved by fusing transcriptional activator or repressor to engineered Cas9 with both catalytic domains inactivated (deadCas9 also known as dCas9) and directed to specific promoter regions [55]. CRISPR offers the opportunity to edit different targets simultaneously [56] and to obtain DNA-free genome edited plants using CRISPR-Cas ribonucleoproteins (RNP) or transient expression systems to deliver DNA cassettes encoding for editing components [57]. Such technology is applied in a wide range of applications spanning from gene silencing and gene insertions to base, RNA, and epigenome editing, therefore allowing programmable editing even of the processes included in the central dogma model [58]. In light of this, researchers have now the capability to fine tune the flow of genetic information across different levels in the central dogma and to act on factors determining the epigenetic memory resulting from plant-environment interactions [59]. Thus, CRISPR represents the best way to introduce or modify genetic information to improve major and minor traits in plants. The advantages offered by CRISPR technologies (easy to adopt, efficiency, specificity) make this technique a valid substitute for any type of gene knock-out or gene insertion technique and direct the large diffusion of its applications in every area of genetic engineering. Furthermore, transgenic and RNAi lines cannot escape from being defined GM organisms, whereas CRISPR lines cannot be assimilated by these rules since the foreign DNA is not necessarily integrated into host cells to produce precise mutations. Indeed, a recently published study of the European Commission regarding the status of new genomic techniques (NGT) under Union law identified limitations to the capacity of the legislation to keep pace with scientific developments, causing implementation challenges and legal uncertainties. It concluded that the applicable legislation is not fit for the purpose of some NGTs and their products and that it needs to be adapted to scientific and technological progress. It may not be justified to apply different levels of regulatory oversight to similar products with similar levels of risk, as it is the case for plants conventionally bred and obtained from certain NGTs.

## 3. Increasing Disease-Resistance in Cereals by Implementing Plant Immunity Through Transgenesis

**Figure 2:**
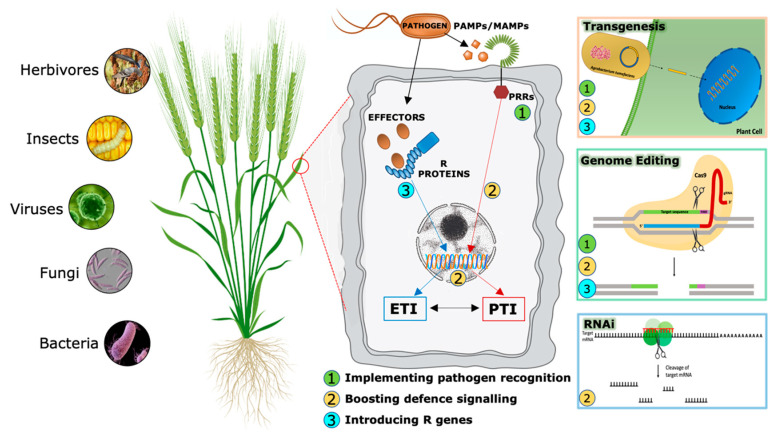
Biotechnological approaches and their possible involvement to enhance cereal resistance to pathogens.

In recent years, significant efforts have been made, and results have been obtained in understanding the interplay between plants and their invaders [60]. During evolutionary warfare with pathogens, plants have evolved sophisticated detection and inducible defense systems to properly defend themselves (Figure 2). Innate immunity is the first step in defense against biotic agents and can be activated within a few minutes after pathogen sensing [61]. The faster pathogen detection occurs, the sooner proper immune responses are mounted by plants, with a consequent higher probability to restrict or block tissue invasion. Therefore, plants deploy hundreds of pattern recognition receptors (PRRs) in the cell plasma membrane, conceptually analogous to Toll-like receptors in animal cells [62], that can identify both non-self-molecules, referred to as pathogen-associated molecular patterns (PAMPs), and altered self-molecules or damage-associated molecular patterns (DAMPs) [63,64]. Ligand binding by its cognate receptor, belonging to the Receptor-Like Kinases (RLKs) or Receptor-Like Proteins (RLPs) classes, triggers the so-called PAMP/DAMP-triggered immunity (P/DTI), which includes, as major downstream signaling events, the calcium influx, a burst of reactive oxygen species (ROS), the activation of downstream signaling pathways leading to gene expression reprogramming, and the production of antimicrobic compounds [65]. A second level of the plant immune system involves plant resistance proteins able to recognize pathogen specific effectors (Avr proteins) and triggers plant defense mechanisms in a more robust way [66]. Plant intracellular immune receptors are nucleotide-binding, leucine-rich repeat receptors (NLRs), which also exist in animals [67]. This kind of resistance is called effector-triggered immunity (ETI) and often induces the hypersensitive response (HR) that includes programmed cell death in infected cells and surrounding areas [68]. Most R genes encode proteins with unique domains that contain a conserved Nucleotide Binding Site called NBS. LRR (Leucin-Rich Repeat) is the second most important domain. NB-LRR receptors may recognize pathogen effectors delivered inside the cell to favor plant colonization [69]. 

Traditionally, PTI and ETI have been considered to act sequentially but independently. However, recent accumulating evidence shows that the distinction between PAMPs and effectors, PRRs and R proteins, therefore between PTI and ETI, cannot strictly be maintained [70,71], suggesting an alternative model in which the two systems interact and share common elements but in which the cellular responses they evoke appear to be distinct. Analyses of specific mutants concluded that the activation of PTI is essential for ETI to function, while ETI can boost the efficiency of PTI and prolong the immune response duration.

Plant hormones, or phytohormones, are naturally occurring signaling compounds with diverse chemical properties. They play critical roles in the adaptation to environmental changes by driving proper responses, including activation of immunity, to a wide variety of biotic and abiotic stresses. The activity of a given hormone depends on its biosynthesis, conjugation, transport, and degradation as well as hormone activation and inactivation [72,73]. Although all hormones regulate several processes independently, inducible defense responses are fine-tuned by very complex crosstalk among hormone signaling outputs [74,75,76]. This enables plants both to adjust their reaction to the type of invader encountered and to efficiently use resources [77]. Interactions between hormonal activities can be either synergistic or antagonistic [78]. Such a complex and multilayered plant immune system offers different levels on which researchers could act through biotechnological approaches in order to enhance or implement plant resistance (Table 1).

### 3.1. Pathogen Detection 

Knowledge of the plant immune system offers the opportunity to develop new strategies of intervention at the pathogen perception level (Table 1). Increased or new recognition ability may be generated in different ways, for example by intra- and interspecies introduction of PRRs from other plants with novel recognition specificity [62,83,84,100,101,102]. In a recent study, the *Arabidopsis thaliana* EF-Tu (elongation factor thermo unstable) receptor, abbreviated as EFR, was transferred to monocot rice to confer resistance to two *Xanthomonas oryzae* pv. O*ryzae* isolates. Rice plants expressing such receptors were able to sense the bacterial ligand of EFR and to elicit an immune response. Moreover, the EFR receptor was able to use components of the rice immune signaling pathway for its function [80]. *AtEFR* was also expressed in wheat [79] driven by the rice actin promoter, and the plants showed enhanced induction of defense-related genes, callose deposition, and resistance against the cereal bacterial pathogen *P. syringae* pv. *Oryzae.* In another study, a lectin receptor-like kinase gene (LecRK) of *Haynaldia villosa*, a diploid wheat relative, has been transferred to wheat variety Yangmai158, which is powdery mildew susceptible [93]. Transgenic wheat plants showed a significant increase in powdery mildew resistance. Moreover, dynamic changes were detected for the expression levels of ROS generating/scavenging genes and marker genes of the salicylic acid (SA) pathway.

A different original approach is represented by engineering novel recombinant PRRs by producing chimeric receptors incorporating the beneficial properties of various RLKs and RLPs [88]; important advances have been achieved, suggesting that the ectodomain of the chimera preserves ligand perception capacity, while the intracellular domain determines the output intensity [80,86,87]. Modular assemblies between Arabidopsis EFR and rice Xa21 [86] have shown that it is reliable to engineer PRRs to increase the amplitude of the induced defense response and to expand the recognition spectrum. Indeed, using the EFR-Xa21 chimera, rice Xa21 kinase domain results functional in Arabidopsis to induce signaling and quantitative immunity against the bacterium *Pseudomonas syringae pv. Tomatoe* and *Agrobacterium tumefaciens*. As rice Xa21 triggers HR-like responses, its intracellular domain has been used to generate chimeric PRR with rice OsCEPiP ectodomain [103]. The related chimera improved cell death following treatment with chitin as well as resistance to the fungal pathogen *Magnaporthe oryzae* [88].

Beyond pathogen-recognition strategies, a better understanding of effectors and their role has allowed interventions at the point of pathogen modulation of host responses. Identification of effector activity targets in plant, for instance, shows which host components are “manipulated” by the invaders to promote disease. In order to interfere with these components of susceptibility, this knowledge was successfully exploited by removing [104,105,106,107] or replacing them with variants that are resistant to the effector activity without losing their native function in the host [108]. For bacterial pathogens expressing transcription activator-like (TAL) effectors that activate the expression of susceptibility genes in the host, resistance can be engineered introducing deletions in the TAL DNA binding sites on the promoter of those genes [89,109]. Another approach to engineer resistance to these bacterial pathogens is to add TAL effector binding sites to a cell-death-promoting (“executor”) gene that is triggered by TAL effectors present in common pathotypes [90,93]. According to information on virulence factor/effector biology, it will be possible to select LRR proteins with new specificities, able to inhibit the growth of necrotrophic or biotrophic pathogens or to target resistance to viruses.

### 3.2. Boosting the Immune Signaling

P/DTI and ETI lead to the activation of the membrane-localized ion channels and an increase in the amount of cytoplasmic calcium. Other early response events include the activation of mitogen-activated protein kinases (MAPKs) [110]. Three hormones are principally involved in downstream signaling pathways caused by P/DTI and ETI: SA, jasmonic acid (JA), and ethylene (ET). Even though SA pathway stimulates resistance to biotrophic and hemibiotrophic pathogens, JA and ET pathways are typically induced upon sensing necrotrophic pathogens and chewing insects [111]. JA and SA have important roles in the activation of transcription factors controlling biotic stress responses, the interplay between different defense signaling pathways, and chemical priming to improve plant resistance through systemic acquired resistance (SAR). However, constitutive induction of SA or JA signaling, besides inducing resistance against pathogens, also leads to pleiotropic negative effects on growth and yield, a process known as growth-defense trade-off which is based on the assumption that plants can allocate resources either to growth or in defense [112]. Activated defense programs require cellular rearrangements at different levels, including machinery involved in transcription, translation, and protein secretion as well as metabolism prioritization of carbon and nitrogen towards production of defense compounds, such as pathogenesis-related (PR) proteins. Such a trade-off represents the output of a complex and fine-tuned phytohormonal crosstalk, and researchers worldwide are trying to unravel key regulatory elements to obtain resistant plants normally growing and producing. Recently, the transcription factor TL1-Binding Factor 1 (TBF1), which is quickly and transiently triggered by pathogen attacks, has been used to produce a “TBF1-cassette” consisting of an immune-inducible promoter and two pathogen-responsive upstream open-reading frames (uORFsTBF1) of the *TBF1* gene. Researchers showed that the utilization of “TBF1-cassette” can enhance broad-spectrum disease resistance with minimal adverse effects on plant growth and development [91]. The timely and tissue localized induction of immunity may prevent the reduction in plant growth and yield, consequences of activated defense responses, thus overcoming the trade-off problem. Moreover, defense responses are controlled by networks of transcriptional regulators [113]. Therefore, the overexpression of specific transcription factors is a potential strategy to engineer resistance, with minimized or no effects on yield. One interesting study concern the rice gene *Ideal Plant Architecture 1* (*IPA1*), known as *OsSPL14*, in which a naturally occurred allelic variant increased yield and resistance to rice blast (Table 1). Specific phosphorylation of IPA1 in response to blast infection alters IPA1 binding specificity. This change in specificity leads the protein to bind to WRKY45, a defense regulator transcription factor, and activate its expression, therefore ensuring quantitative resistance to the pathogen [93].

### 3.3. R Gene Transfer 

Adult plant resistance (APR) or “slow rusting” wheat genes represent a class of potential transferable *R* genes [114]. Different APR genes are known, but only two, *Lr34* and *Lr67* (Table 1)*,* have been cloned [115,116]. *Lr34* encodes an ATP-binding cassette (ABC) transporter with an unknown substrate. Transgenic wheat lines expressing *Lr34* gene displayed enhanced resistance to multiple biotrophic pathogens including the leaf rust pathogen and powdery mildew both at seedling and adult stages [94,117]. Similarly, the wheat *Lr67* resistance gene is a specific dominant allele of a hexose transporter that provides resistance to powdery mildew and multiple rusts. Introduction of the *Lr34* allele by transformation into rice [95], barley [94], sorghum [96], maize [97], and durum wheat [98] and of *Lr67* into barley [99] produced resistance to a broad spectrum of biotrophic pathogens such as *Puccinia triticina* (wheat leaf rust), *P. striiformis* f. sp. *Tritici* (stripe rust), *P. graminis* f. sp. *Tritici* (stem rust), *Blumeria graminis* f. sp. *Tritici* (powdery mildew), *P. hordei* (barley leaf rust) and *B. graminis* f. sp. *Hordei* (barley powdery mildew), *Magnaporthe oryzae* (rice blast), *P. sorghi* (maize rust), and *Exserohilum turcicum* (northern corn leaf blight) [94,95,97]. The mechanism by which resistance is triggered by *Lr34* and *Lr67* is poorly understood, although it is likely that it provides the activation of biotic or abiotic stress responses allowing the host to limit pathogen development and growth.

Wheat resistance to Fusarium species has been greatly improved by expressing either a barley uridine diphosphate-dependent glucosyltransferases (UGT), *HvUGT13248*, involved in mycotoxin detoxification [118], or pyramided inhibitors of cell wall-degrading enzymes secreted by the fungi, such as the bean polygalacturonase inhibiting protein (*PvPGIP*2) and TAXI-III, a xylanase inhibitor [119]. Interestingly, greater resistance to *Fusarium graminearum* has been observed in wheat plants simultaneously expressing the *PvPGIP*2 in lemma, palea, rachis, and anthers, whereas the expression of this inhibitor only in the endosperm did not affect FHB symptom development, hinting that further spread of the pathogen in wheat tissues no longer can be blocked once it reaches the endosperm [120].

## 4. Increasing Disease-Resistance in Cereals by Using Gene Expression or Editing Techniques

### 4.1. RNA Interference (RNAi)

RNA interference (RNAi) was first discovered in plants as a molecular mechanism involved in the recognition and degradation of non-self-nucleic acids, principally directed against virus-derived sequences. In addition to its defensive role, RNAi is essential for endogenous gene expression regulation [121]. Initiation of RNAi occurs after double-stranded RNAs (dsRNAs) or endogenous microRNAs are processed by Dicer-like proteins. The resulting small interfering (si)RNAs can be recruited by Argonaute (AGO) proteins that recognize and cleave complementary strands of RNA, resulting in gene silencing. RNAi-based resistance can be engineered against many viruses by expressing “hairpin” structures, double-stranded RNA molecules that contain viral sequences, or simply by overexpressing dysfunctional viral genes [122]. Moreover, a single double-stranded RNA molecule can be processed into a variety of siRNAs and thereby effectively target several virus sequences using a single hairpin construct. 

Over the last two decades, RNAi has emerged as a powerful genetic tool for scientific research. In addition to basic studies on the determination of gene function, RNA-silencing technology has been used to develop plants with increased resistance to biotic stresses (Figure 2), (Table 2) [123,124]. 

Indeed, the impact of RNAi technology deployed as a GM solution against viruses is clearly demonstrated in different studies [125,126,127]. Wheat dwarf virus (WDV) is a member of the Mastrevirus genus of the *Geminiviridae* family. This virus translates four viral proteins and causes economical losses in wheat and barley when it is transmitted to plants through leafhoppers. Kis et al. [126] targeted 13 different wheat- and barley-infecting WDV strains to identify conservative target sites and design miRNAs by using the miRNA precursor (hvu-MIR171) backbone of barley. They constructed a polycistronic artificial microRNA (amiRNA) precursor, which expresses three amiRNAs at the same time. As a result, transgenic barely plants that express amiRNAs at high levels presented no infection symptoms.

Recently, RNAi has been explored as a strategy to also control fungi and oomycetes. Fungal target genes are obvious candidates for this approach, as disruption is known to be lethal. A biotechnological method, termed host-induced gene silencing (HIGS), has emerged as a promising alternative in plant protection because it combines high selectivity for the target pathogen with minimal side effects, as compared with chemical treatments. Significant effects have been observed in transgenic Arabidopsis and barley (*Hordeum vulgare*) plants, expressing *via* HIGS a 791 nucleotide (nt) dsRNA (CYP3RNA) targeting all three *CYP51* genes (*FgCYP51A*, *FgCYP51B*, *FgCYP51C*) of *Fusarium graminearum* (*Fg*) that led to the inhibition of fungal infection [128].

Cheng et al. [129] reported that the expression of RNAi sequences derived from an essential *Fg* virulence gene, the chitin synthase 3b (*Chs3b*), is an effective method to enhance resistance of wheat plants against fungal pathogens. Three hairpin RNAi constructs corresponding to the different regions of *Chs3b* were found to silence *Chs3b* in *Fg* strains. Co-expression of these three RNAi constructs in two independent elite wheat cultivar transgenic lines conferred high levels of stable and consistent resistance (combined type I and II resistance) to both Fusarium Head Blight (FHB) and Fusarium Seedling Blight (FSB).

A better understanding of this process in diverse plant-pathogen interactions may allow to better optimize HIGS strategies providing field-relevant levels of resistance [130,131,132]. In short, RNAi appears to be a promising additional control strategy in the arsenal of plant breeders against at least some pathogens. The modular nature of RNAi is especially suitable for multiplexing *via* synthetic biology approaches. In addition, RNAi strategies may be particularly relevant when no pathogen resistance can be identified in natural populations.

### 4.2. CRISPR/Cas9 Mediated Genome Editing

In plant research, NBTs are attracting a lot of attention. NBTs appear to be suitable for many different fields in plant science, such as developmental processes and adaptation/resistance to (a)biotic stresses [133]. NBTs include the most recent and powerful molecular approaches for precise genetic modifications of single or multiple gene targets. They employ site-directed nucleases to introduce double-strand breaks at predetermined sites in DNA.

The rapid increase in scientific publications documenting the use of CRISPR/Cas highlights how this technique has a greater success rate in gene modification compared to the other available nucleases. Actually, the application of CRISPR/Cas technologies to edit plant genomes is proving to be a powerful tool for future enhancement of agronomic traits in crops, qualitative and health parameters, tolerance to abiotic stress [134], and also for the improvement of biotic stress resistance (Table 2) [135]. 

In a recent study, *MLO* loci have been targeted by RNA-guided Cas9 endonuclease in bread wheat [136]. *MLO* encodes a protein with seven transmembrane domains localized in the plasma membrane and is ubiquitously present in monocots and dicots [36]. It had previously been reported that *MLO* were susceptibility genes and that homozygous loss-of-function mutants had significantly increased resistance to powdery mildew in barley, *Arabidopsis,* and tomato [141,142,143]. Bread wheat plants mutated by CRISPR/Cas9 in one (*TaMLO-A1*) of the three *MLO* homeoalleles showed improved resistance to *Blumeria graminis* f. sp. *tritici* infection, a finding that once again demonstrated the important role of *TaMLO* genes in powdery mildew disease [136]. Another example of CRISPR/Cas9-derived resistance against the same disease is the knockout of *TaEDR1* [43], conferring resistance to powdery mildew in wheat. Recently, Su et al. [140] have reported that *TaHRC*, a gene that encodes a putative histidine-rich calcium-binding protein, is the key determinant of resistance to FHB. Authors have demonstrated that *TaHRC* encodes a nuclear protein conferring FHB susceptibility and that a CRISPR–Cas9-mediated deletion spanning the start codon of this gene results in FHB resistance. Plant mutants had significantly lower FHB severity than their wild type, suggesting that *TaHRC* affects FHB susceptibility and that loss of function of *TaHRC* confers Fhb1 resistance. Plants resistant to rice blast disease were generated through CRISPR/Cas9-mediated disruption of *OsERF922* and *OsSEC3A* genes in rice [138,139]. *Ossec3a* mutant plants in a putative subunit of a complex involved in exocytosis revealed a pleiotropic phenotype including improved resistance against *Magnaporthe oryzae*, higher levels of SA and its related genes, but also dwarf stature [138]. In contrast, no alteration of different agronomic traits was observed in T_1_ and T_2_ transgene free plants mutated in the ET responsive factor (ERF) 922, a transcription factor involved in multiple stress responses. Mutant plants had a reduced number of blast lesions at both seedling and tillering stages [139]. 

Relatively few studies have been published on the application of the CRISPR/Cas systems to counteract crop bacterial diseases. CRISPR/Cas9 editing of *OsSWEET13* has been performed in rice to achieve resistance to bacterial blight disease caused by bacterium *Xanthomonas oryzae* pv. *oryzae* [137]. *OsSWEET13* is a susceptibility gene encoding a sucrose transporter involved in plant-pathogen interaction. *X. oryzae* produces an effector protein, PthXo2, which induces *OsSWEET13* expression in the host and the consequent condition of susceptibility. Zhou et al. [137] obtained a null mutation in *OsSWEET13* in order to better explore PthXo2-dependent disease susceptibility, and resultant mutants were resistant to bacterial blight. Further genome editing strategies for multiplexed recessive resistance using a combination of the major effectors and other *R* genes will be the next step toward achieving bacterial blight resistance.

## 5. Conclusions and Perspectives

Our planet is facing unprecedented challenges because of a rising and more affluent world population, while almost half of global cereal production is lost to diseases, biodiversity is diminishing at an alarming pace, and the average temperature on earth continues to rise. To meet the global challenge of food availability, we will need to shift our mentality and lifestyle, increase investments in knowledge creation, and facilitate the usage of innovative technologies, which can shorten timings to reach these goals. On the other hand, agriculture and food production must become more sustainable. The environmental footprint of agriculture needs to diminish, and farming must adapt to the rapidly changing climate. Given that we are witnessing how these aspects are threatening crop yields worldwide, all possible approaches are required to meet these challenges. Mutagenesis had, has and will certainly have an impact on crop genetics and breeding in the attempt to increase stress resistance and productivity. The ability of the DNA to mutate provides genetic variability, which is basilar for plants, as for all organisms, to evolve and adapt to environmental changes. New genotypes with new traits, likely of agronomic interest, can be created in cultivated plants by artificially inducing mutations in their genomes. Crops with improved characteristics can be obtained by both transgenesis and conventional plant breeding, respectively by adding a new gene to the genome of a crop plant or by crossing plants with desirable characteristics and selecting combinations of genes inherited from the two parent lines. With respect to when transgenesis technology was developed, our knowledge on plant gene function(s) and activity has drastically increased, satisfying one prerequisite for transgenesis to be preferable, especially in case the gene of interest do not exist in a species that can be successfully crossed with the crop. Secondly, modern high yielding crop varieties mostly result from careful selection of lines with specific combinations of value-added genes; such combination might be destroyed in the attempt to add by crossing a useful gene variant that has been newly discovered in a wild relative. Nonetheless, additional genes, closely located in the genome to the gene of interest, are almost always transferred as well. It will take several years and different generations to restore those gene combinations, even when using modern molecular breeding techniques, such as marker assisted breeding. These problems can be avoided by introducing a new added-value gene into the high yield crop by direct transformation, therefore by transgenesis. For instance, plant cultivars with *R* genes can be created by a transgenic approach rather than by a traditional crossing approach. An additional advantage of transgenic strategies is that linkage drag can be prevented. In resistance breeding, linkage drag refers to the undesirable reduction in crop yield and quality sometimes associated with selection of genetic resistance to disease. One of the most efficient and sustainable solutions to control plant resistance to pathogens is to use genetic modification and genome editing techniques to complement and extend modern breeding efforts. 

Genome engineering techniques have made important advances over the last decades, allowing the capability not only to control but even to edit gene expression in a precise and secure manner, see Table 1 and Table 2. Genome editing allows scientists to mutate the genome of plants in a manner similar to how mutation occurs in nature, generating heritable mutations in a predictable trait-related genomic location and thus creating a series of variable phenotypes for breeding within a single generation. 

The application of such biotechnological techniques in agriculture can potentially improve food availability and security by raising crop resistance to pathogens, adverse weather and soil conditions, by enhancing the adaptability of crops to different climates and by improving yields, particularly of staple food crops such as cereals (Figure 2). Biotechnology could, over the next two decades, deliver the next wave of technological change; change that could be fundamental in understanding the molecular basis of disease resistance in enough detail to make precise predictions about engineering plants to express resistance proteins that can either recognize pathogen molecules essential for pathogenicity or finely tune hormone signaling for the benefit of crop yield [144]. In this manner, it is anticipated that biotechnological approaches can engineer durable disease resistance in crops. Examples of genetic disease solutions currently available for bacterial, viral and fungal pathogens are listed in Table 1 and Table 2.

An ambitious target for the future is to continue combining science-based knowledge with biotechnological methods to develop plants that have higher resilience to (a)biotic stresses. This will enable farmers to produce high yields while decreasing the use of chemicals and water.

## Figures and Tables

**Table 1:** Biotechnological interventions to increase disease resistance in cereals.

Immunity Level of Intervention	Biotechnological Intervention	Gene	Species	Enhanced Resistance to	References
*Pathogen sensing*	Interspecies/interfamily transfer of known PRRs	*AtEFR*	Wheat	*Pseudomonas syringae* pv. *oryzae*	[79]
		*AtEFR*	Rice	*Xanthomonas oryzae* pv. *oryzae-*derived *elf18*	[80]
		*AtEFR*	Rice	*Acidovorax avenae* subsp. *avenae*	[81]
		*OsXa21*	Rice	*Xanthomonas oryzae* pv. *oryzae*	[82]
		*TaRLK1* and *TaRLK2*	Wheat	*Blumeria graminis* f. sp. t*ritici*	[83]
		*HvLEMK1*	Barely, Wheat	*Blumeria graminis f.sp. hordei; Blumeria graminis* f. sp. t*ritici*	[84]
		*HvLecRK-V*	Wheat	*Blumeria graminis* f. sp. t*ritici*	[85]
	Production of chimeric receptor kinases and *R* genes	*AtEFR-OsXa21*	Rice	*Pseudomonas syringae pv. tomato; Agrobacterium tumefaciens; Xanthomonas oryzae pv. oryzae*	[86,87]
		*OsXa21-OsCEPiP*	Rice	*Magnaporthe oryzae*	[88]
*Effector detection*	Deletion of effector binding sites	*Os11N3/OsSWEET14*	Rice	*Xanthomonas oryzae pv. oryzae*	[89]
	Addition of effector binding sites	OsXa27	Rice	*Xanthomonas oryzae pv. oryzae*	[90]
*Immune signaling*	Altered expression of signaling components	*AtNPR1*	Rice	Broad-spectrum of pathogens	[91]
	Altered expression of transcription factors	*TaPIMP1*	Wheat	*Bipolaris sorokiniana*	[92]
		*OsIPA1/OsSPL14*	Rice	*Magnaporthe oryzae*	[93]
*R genes*	Transfer of APR alleles	*TaLr34*	Barely, Rice, Sorghum Maize, Durum wheat	Multiple biotrophic pathogens	[94,95,96,97,98]
		*TaLr67*	Barely	Multiple rusts and powdery mildew	[99]

**Table 2:** Examples of gene expression or editing techniques to increase disease resistance in cereals.

Molecular Technique	Biotechnological Intervention	Gene	Species	Enhanced Resistance to	References
*RNAi*	Viral gene silencing	Wheat streak mosaic virus genes	Wheat	Wheat streak mosaic virus (WSMV)	[125]
		Wheat dwarf virus genes	Barely	Wheat dwarf virus (WDV)	[126]
	Host-induced gene silencing	*FgCYP51A*, *FgCYP51B* and *FgCYP51C*	Barely	*Fusarium graminearum*	[128]
		*FgCh3b*	Wheat	*Fusarium graminearum*	[129]
		*PtMAPK1*, *PtCYC1, PtCNB*	Wheat	*Puccinia triticina, P. graminis* and *P. striiformis*	[130,131]
		*FcGls*	Wheat	*Fusarium culmorum*	[132]
*CRISPR/Cas9*	Silencing of host genes	*TaMlo-A1*	Wheat	*Blumeria graminis* f. sp. t*ritici*	[136]
		*OsSWEET13*	Rice	*Xanthomonas oryzae pv. oryzae*	[137]
		*OsERF922*	Rice	*Magnaporthe oryzae*	[138]
		*TaEDR1*	Wheat	*Blumeria graminis f. sp. tritici*	[43]
		*OsSEC3A*	Rice	*Magnaporthe oryzae*	[139]
		*TaLpx-1*	Wheat	*Fusarium graminearum*	[102]
		*TaHRC*	Wheat	*Fusarium graminearum*	[140]

## Data Availability

Not applicable.
